# A Novel Amphibian Antimicrobial Peptide, Phylloseptin-PV1, Exhibits Effective Anti-*staphylococcal* Activity Without Inducing Either Hepatic or Renal Toxicity in Mice

**DOI:** 10.3389/fmicb.2020.565158

**Published:** 2020-10-26

**Authors:** Yue Liu, Daning Shi, Jin Wang, Xiaoling Chen, Mei Zhou, Xinping Xi, Jianming Cheng, Chengbang Ma, Tianbao Chen, Chris Shaw, Lei Wang

**Affiliations:** ^1^College of Pharmacy, Nanjing University of Chinese Medicine, Nanjing, China; ^2^Natural Drug Discovery Group, School of Pharmacy, Queen’s University Belfast, Belfast, United Kingdom; ^3^School of Government, Peking University, Beijing, China; ^4^Department of Chinese Medicine, Pizhou People’s Hospital, Pizhou, China

**Keywords:** antimicrobial peptide, phylloseptin, anti-*staphylococcal* activity, infected mouse, toxicity, amphibian skin

## Abstract

In order to part address the problem of drug-resistant pathogens, antimicrobial peptides (AMPs) have been proposed as alternatives to traditional antibiotics. Herein, a novel phylloseptin peptide, named phylloseptin-PV1 (PPV1), is described from the defensive skin secretion of the Neotropical white-lined leaf frog, *Phyllomedusa vaillantii*. The peptide was synthesized by solid phase peptide synthesis (SPPS) and purified by RP-HPLC, prior to assessment of its biological activities. PPV1 not only demonstrated potent antimicrobial activity against planktonic ESKAPE microorganisms and the yeast, *Candida albicans*, but also inhibited and eradicated *Staphylococcus aureus* and MRSA biofilms. The antimicrobial mechanism was shown to include permeabilization of target cell membranes. The *in vivo* antimicrobial activity of the peptide was then evaluated using mice. PPV1 also exhibited antiproliferative activity against the cancer cell lines, H157, MCF-7, and U251MG, but had a lower potency against the normal cell line, HMEC-1. Although, the peptide possessed a moderate hemolytic action on mammalian red blood cells *in vitro*, it did not induce significant hepatic or renal toxicity in injected infected mice. These studies have thus found PPV1 to be a potent phylloseptin group AMP, which can effectively inhibit staphylococci, both *in vitro* and *in vivo*, without eliciting toxicity. These data thus provide support for further evaluation of PPV1 as a novel antimicrobial agent with therapeutic potential.

## Introduction

The discovery and utilization of antibiotics in the last century have undoubtedly saved countless lives. However, with the widespread use and abuse of these therapeutics, resistant microorganisms have emerged and developed into a serious issue in the clinic. As one of the ESKAPE pathogens, *Staphylococcus aureus* is widely distributed and is very easily spread among the population by simple contact with patients or with their belongings (Imperial and Ibana, 2016). It only causes mild symptoms in most people, but it also induces severe infections in people with other serious health problems (Ansari et al., 2019). It has already been proven to be one of the most common nosocomial infections (Dreyfus et al., 2020), and can cause pneumonia transmitted through ventilators, surgical site infections, and catheter-associated bloodstream infections (Kwiecinski and Horswill, 2020). The emergence of resistance to conventional antibiotics, for instance, methicillin-resistant *S. aureus* (MRSA), has resulted in an increase in severe infections, even death, and this much more frequent than infections with a non-resistant form of the pathogen. Resistance to first-line drugs, such as vancomycin, which treat *S. aureus* infections effectively, has made patients unable to cope with MRSA infections (McGuinness et al., 2017). Therefore, there is an urgent need to discover new antibiotic alternatives to tackle the global problem.

In Nature, antimicrobial peptides (AMPs) are considered to be the first line of defense against viruses, bacteria, and fungi, and they play an important role for most living organisms in their defense against pathogens (Radek and Gallo, 2007). Their broad-spectrum antimicrobial activity, prevention of biofilm formation and fast killing effects, have also attracted much attention among medical researchers (Gao et al., 2017; Molchanova et al., 2017). So far, more than 3000 AMPs have been discovered, among which more than one third have been isolated from the skin secretions of amphibians (Yang et al., 2016). These AMPs are widely distributed among different frog species and classified into several families based on their structural characteristics. The most famous amphibian derived AMP is magainin that was discovered from the skin secretion of *Xenopus laevis* (Zasloff, 2019), and it is the first AMP derived from amphibian which has been investigated in the Phase III clinical trial (ClinicalTrials.gov Identifier: NCT01590758). Besides, other AMPs, such brevinin (Timmons et al., 2019), temporin (Gaiser et al., 2019), ranatuerin (Zhou et al., 2019), and dermaseptin (Ying et al., 2019), also demonstrated outstanding effects in the recent studies.

Among all of the AMPs derived from amphibian skin, phylloseptin is one of the AMP families isolated from the skin secretion of *Phyllomedusa* frogs (Zhang et al., 2010), shown to have effective antimicrobial activity (Leite et al., 2005). These peptides most often contain 19 amino acid residues with a highly conserved N-terminal domain, FLSLIP-, and C-terminal amidation (Wan et al., 2015). Phylloseptins usually have an electrostatic interaction between their positively charged residues and the negatively charged molecules on the cell envelopes of microorganisms, such as lipopolysaccharides (LPS), teichoic acid, and negatively charged phospholipids (Resende et al., 2008). Then, they are also able to form an α-helix conformation in target cell membrane environments and to permeabilize these in the microorganisms (Dathe and Wieprecht, 1999).

Phylloseptins have been proven to be effective against many bacteria as well as fungi and parasites (Raja et al., 2013). Particularly, they exhibit potent antimicrobial activity against Gram-positive bacteria, even those showing resistance to conventional antibiotics (Chung and Khanum, 2017). However, the evaluation of the antimicrobial potency of phylloseptins has only been conducted at the cellular level. Using animal models for phylloseptins is fast becoming essential for further elucidating their potential and efficacy as new drug candidates. Here, the discovery of a novel phylloseptin from the defensive skin secretion of the white-lined leaf frog, *Phyllomedusa vaillantii*, is described following use of “shotgun” cloning and mass spectrometry. This peptide was then synthesized and found to exhibit broad-spectrum antimicrobial activity against several bacteria. In addition, since *in vitro* studies cannot address the complex reactions which occur to cause antimicrobial effects, two of the most common inbred and outbred mouse strains, C57BL/6 J and CD1, were chosen as models for studying peptide *in vivo* biofunctional activity. PPV1 not only exhibited antimicrobial activity against *S. aureus* in mice, but showed low toxicity *in vivo* as well.

## Materials and Methods

### Acquisition of Skin Secretion From *Phyllomedusa vaillantii*

Skin secretion of *P. vaillantii* was purchased from Mr. Juan Chavez Lopez, (Peru Biotech E.I.R.L., Santiago de Surco, Peru). The specimens were washed gently with deionized water and their skins were stimulated by gentle electrical stimulation (5 V, 100 Hz, 140 ms pulse width) (Tyler et al., 1992). Skin secretions were washed from the skin with deionized water into chilled containers, snap frozen in liquid nitrogen and immediately lyophilized. The study was performed according to guidelines given in the United Kingdom Animal (Scientific Procedures) Act 1986, project license PPL 2694, issued by the Department of Health, Social Services and Public Safety, Northern Ireland. Procedures had been vetted by the IACUC of Queen’s University Belfast, and approved on 1st March, 2011.

### “Shotgun” Cloning of A *Phyllomedusa vaillantii* Skin Secretion-Derived cDNA Library

The shotgun cloning was performed as described in detail in a previous study (18). Briefly, poly-A mRNA was extracted from the lyophilized skin secretion using a Dynabeads mRNA Direct kit (Dynal Biotech, United Kingdom), and made into a first-strand cDNA library. RACE-PCR was performed to obtain full length nucleotide sequences using a SMART-RACE Kit (Clontech, Palo Alto, CA, United States) and a degenerate sense primer (S1; 5′-ACTTTCYGAWTTRYAAGMCCAAABATG-3′ (Y = C/T, W = A/T, R = A/G, M = A/C, B = T/C/G), which was designed to a highly conserved segment of the signal peptide-encoding domain of cDNAs cloned previously from other *Phyllomedusa* frogs (19). The PCR products were cloned using a pGEM^®^-T Easy Vector system (Promega Corporation, Southampton, United Kingdom), and sequenced by an ABI 3100 automated capillary sequencer (Applied Biosystems, Foster City, CA, United States).

### LC-MS Analysis of Peptides in the Skin Secretion

Five milligrams of lyophilized skin secretion were dissolved in 1 ml of trifluoroacetic acid (TFA)/water (0.05/99.95, v/v). Then 20 μl was injected into an LC-MS (LCQ fleet ion trap Mass spectrometry) fitted with a C18 column (4.6 × 250 mm, Jupiter) and eluted at a rate of 0.5 ml/min. The sample peptides were separated by gradient elution from 100% TFA/water (0.05/99.95, v/v) to 100% ACN/TFA/water (80.00/0.05/19.95, v/v/v) in 240 min. The sheath gas pressure was set to 20 psi and auxiliary gas flow rate was set to five arbitrary units. The spray and capillary voltage were set to 4.5 kV and 49 V separately, while the capillary temperature was set to 275°C. The total ion chromatograph (TIC) was obtained and the two most abundant peaks were further selected and subjected to MS/MS fragmentation with a normalized collision energy (NCE) of 30. Then the spectra were searched against the translated amino acid sequences from “shot-gun” cloning by the Proteome Discoverer 1.0. Finally, the mass, amino acid sequence, and post-translational modifications of the novel peptide, were obtained.

### Solid Phase Peptide Synthesis

The mature peptide was chemically synthesized by Fmoc chemistry using a Tribute automated solid-phase synthesizer (Protein Technologies, Inc., Tucson, AZ, United States), which was described in detail in a previous study. Briefly, Fmoc groups were removed to free the α-amine by using piperidine/DMF (20/80, v/v) solution. Then, the peptide bond was coupled in the presence of HBTU and 1M N-methylmorpholine. After this, the final Fmoc group was removed from the peptide chain and the resin and side chain protection groups were further removed by use of a cleavage cocktail, consisting of 94% TFA, 2% 1, 2-ethanedithiol, 2% (v/v) thioanisole, and 2% (v/v) water. The peptide was precipitated using ice-cold ether and lyophilized. The crude peptide was further purified by RP-HPLC (Cecil Adept HPLC, coupled with C18 column 21 × 250 mm) and the molecular mass was determined by MALDI-TOF mass spectrometer (Voyager DE MALDI-TOF-MS, Applied Biosystems).

### Secondary Structure Analysis

The secondary structure of the peptide was determined by use of circular dichroism spectrometry (JASCO J-815 CD spectrometer, Jasco, Essex, United Kingdom) as described in a previous study (Gao et al., 2016; Wu et al., 2016). Peptides were dissolved in 10 mM ammonium acetate buffer or 50% TFE in 10 mM ammonium acetate buffer at a final concentration of 100 μM. The percentage of the α-helix structure was predicted by the online tool K2D3 (Louis-Jeune et al., 2012). Peptide samples were measured within the range of 190–250 nm at 20°C. The parameters were set as: 200 nm/min scanning speed, a bandwidth of 1 nm, and 0.5 nm data pitch.

### Antimicrobial Susceptibility Assays

The antimicrobial activity of the peptide was evaluated in minimum inhibitory concentration (MIC) and minimum bactericidal concentration (MBC) assays utilizing the broth dilution method as in a previous study (Gao et al., 2016). Microorganisms used included Gram-positive bacteria, *S. aureus* (NCTC 10788 and ATCC 6538), Methicillin-resistant *S. aureus* (MRSA, ATCC 12493) and *Enterococcus faecalis* (NCTC 12697), Gram-negative bacteria, *Escherichia coli* (NCTC 10418 and ATCC CRM-8739), *Pseudomonas aeruginosa* (ATCC 27853 and ATCC CRM-9027), and *Klebsiella pneumoniae* (ATCC 43816) and the yeast *Candida albicans* (NCYC 1467). Besides, we employed the antibiotic resistance strains from ATCC KPC Panel (ATCC MP-24), including *E. coli* (ATCC BAA-2340) and *K. pneumoniae* (ATCC BAA-1705 and BAA-2342). The clinic isolates, *S. aureus* (B038 V1S1A and B042 V2E1A), and *P. aeruginosa* (B004 V2 S2 B), from cystic fibrosis patients were also tested. The antibiotics resistance profiles for these three strains were showed in Supplementary Table 1. The microorganism treated with broth media was used as the negative control. Vancomycin (for Gram-positive bacteria), gentamicin (for Gram-negative bacteria) and amphotericin B (for *C. albicans*) were employed as the positive controls. After performing the MIC assays, 10 μl of the medium from each well were inoculated onto a Mueller-Hinton agar (MHA) plate and incubated for 24 h. The lowest concentrations that showed no evidence of colony growth were considered as the MBCs. For investigating the thermal stability of the peptide, it was pre-incubated at temperatures of 20, 40, 60, 80, and 100°C, for 30 min before testing.

### Antibiofilm Assays

The minimal biofilm inhibition concentration (MBIC) and the minimal biofilm eradication concentration (MBEC) assays were performed as previously described with some slight modifications (Gao et al., 2016).

For the MBIC assay, the peptide stock solutions were prepared in the same way as for the MIC assays. A 10^6^ CFU/ml bacteria suspension in TSB (for Gram-positive bacteria) or LB (for Gram-negative bacteria) was mixed with corresponding peptide solutions in a 96 well plate and incubated at 37°C for 24 h. Then, the culturing medium in the wells was discarded and the well of the plate was washed with 100 μl PBS and fixed with 100 μl methanol for 30 min. The biofilm was air-dried and subsequently stained with 0.1% (w/v) crystal violet for 30 min and then further washed with PBS. Thereafter the crystal violet was solubilized in 100 μl 33% acetic acid and the absorbance at 595 nm was measured with a Synergy HT plate reader (BioTek, United States).

For the MBEC assay, 100 μl of the same diluted inoculum were dispensed into each well of a 96-well plate for 24–48 h to form mature biofilms. Then mature biofilms were washed with PBS to remove the planktonic bacteria. The biofilm was then treated with peptide solutions at 37°C for 24 h. After this, the plate was washed and stained as above. For both MBIC and MBEC assays, biofilm in the broth media was used as the negative control. A cocktail containing 1% SDS, 1% EDTA, 1% NaOH, and 0.1% NaClO was used as the positive control (Stiefel et al., 2016).

### Time-Killing Assays

The kinetic time-killing assays were performed using *S. aureus* (NCTC 10788), *E. coli* (NCTC 10418), and MRSA (ATCC 12493). A suspension culture (10^5^ CFU/ml) of each bacteria was mixed with peptide solutions at the concentration of respective MIC and 4 × MIC. Then, the aliquots were removed from culture tubes at 0, 5, 10, 15, 30, 60, 90, 120, and 180 min intervals. The bacteria were seeded onto MHA plates and incubated at 37°C for 24 h prior to colony counting.

### Membrane Permeability Assays

Membrane permeability assays were carried out using SYTOX Green Nucleic Acid Stain as in a previous study (Huang et al., 2017). Bacterial cells in log-phase were harvested and resuspended in 5% TSB in 0.85% NaCl solution to achieve the concentration of 1 × 10^8^ CFU/ml. Peptide solutions were mixed with bacterial suspension and 5 μM SYTOX green dye in the wells of a black 96 well plate. The positive control employed the permeabilized bacterial cell suspension that had been damaged by 70% isopropanol. In addition, the well-known cell lytic peptide, melittin, was also tested for the comparison. The black plate was incubated for 2 h at 37°C in the dark. The SYTOX green dye could bind with nucleoid DNA when the cell membrane was compromised. The fluorescent intensity of each well was recorded using an ELISA plate reader (Biolise BioTek EL808, Winooski, VT, United States) with excitation and emission wavelengths set to 485 and 528 nm, respectively.

### Screening of Antiproliferative Activity

Breast cancer (MCF7, ATCC HTB-22), human neuronal glioblastoma (U251MG, ECACC General Cell Collection: 09063001), non-small cell lung cancer (NCI-H157, ATCC CRL-5802), and dermal microvascular endothelial (HMEC-1, ATCC CRL-3243) cell lines were seeded at a density of 5 × 10^3^ cells per well onto 96-well plates. Each cell line was treated with peptide and incubated for 24 h. Ten microliters of 5 mg/mL MTT solution were added and incubated for 4 h. The supernatants were removed and 100 μL of DMSO solution were added into all wells to dissolve the formazan crystals. The Synergy HT plate reader (BioTek, Winooski, VT, United States) set at 550 nm, was used to record the absorbance.

### Hemolysis Assays

The hemolytic activity of the peptide was assessed as previously described with some modifications (Gao et al., 2016; Wu et al., 2016). Peptides were incubated with a 2% horse red blood cell suspension in a final concentration range from 1 to 512 μM, and all the tested samples were kept at a constant 37°C for 2 h. The negative controls employed were PBS while the positive controls included the non-ionic detergent, 1% Triton X-100 (Sigma-Aldrich, St. Louis, MO, United States). The sample supernatants were used to assess the extent of hemolysis by measuring their OD values at 570 nm.

### Antimicrobial Activity of the Peptide in *S. aureus* Infected Mice

A suspension of *S. aureus* (1 × 10^8^ CFU/ml in 0.1 ml PBS), was injected into the peritoneal cavity of female C57BL/6 J mice (6–8 weeks old) to initiate infection. Mice were treated with peptide at 5 μg/g and vancomycin at 50 μg/g by intraperitoneal injection, respectively, at 2 h post-infection (10 mice for each group). Mice were monitored for 48 h and euthanized promptly if they became moribund (Mourtada et al., 2019). The livers and kidneys were then harvested and embedded in paraffin, sectioned, mounted on glass slides and stained with hematoxylin and eosin (H&E). All procedures involving animals were approved by the Animal Care and Use Committee of Nanjing University of Chinese Medicine and performed strictly according to the Guide for the Care and Use of Laboratory Animals (Ethics review approval number: ACU191002).

### Determination of *In vivo* Toxicity of Peptide

Ten CD-1 mice (male, 6–8 weeks old) were dosed with peptide at 5 μg/g twice daily for 8 days by intraperitoneal injection of peptide dissolved in PBS. The second daily dose was administered approximately 8 h after the first dose. Another 10 mice treated with PBS only were employed as negative control. On day 9, blood was withdrawn for evaluation of RBC parameters and serum chemistries, and the animals were euthanized for necropsy and histologic analyses (Mourtada et al., 2019). Harvested tissues were embedded in paraffin, sectioned, mounted on glass slides and stained with H&E. All procedures involving animals were approved by the Animal Care and Use Committee of Nanjing University of Chinese Medicine and performed strictly according to the Guide for the Care and Use of Laboratory Animals (Ethics review approval number: ACU191002).

### Statistical Analyses

For antimicrobial and antibiofilm assays, all experiments were repeated at least three independent experiments and the MICs, MBCs, MBICs, and MBECs were determined as the lowest concentration with no significance to the positive control via unpaired two-tailed Student’s *t*-test. One-way ANOVA was employed for membrane permeability assay, MTT test and hemolysis assay. The survival rates of mice were analyzed by log rank test, and the serum tests were compared using unpaired two-tailed Student’s *t*-test (GraphPad Prism 6.01, GraphPad Prism Inc., La Jolla, CA, United States).

## Results

### Identification of Phylloseptin-PV1 From Skin Secretion

**FIGURE 1:**
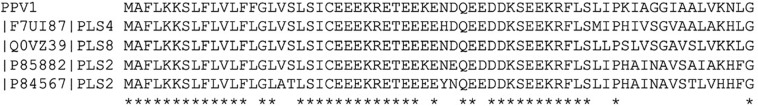
The alignment of translated open-reading frame amino acid sequences of the phylloseptin-PV1 precursor and the top four similar hits from the Uniprot database. The identical amino acids are indicated by asterisks.

A novel peptide-encoding cDNA was repeatedly isolated from the skin secretion cDNA library of the white-lined leaf frog, *P. vaillantii*. The translated open-reading frame (ORF) amino acid sequence consists of 66 amino acid residues, including a putative signal peptide of 22 amino acid residues (Supplementary Figure 1). The alignment of proprepeptides showed that the phylloseptins from different species share a highly conserved propeptide domain, while the mature peptide region exhibits high degrees of variation (Figure 1). However, all phylloseptin proprepeptides contain a Gly residue at the C-terminus which acts as an amide donor. The presence of the cDNA-encoded mature peptide in the skin secretion was confirmed by MS/MS fragmentation and it also confirmed the presence of the post-translational modification of C-terminal amidation (Supplementary Figure 2). As this peptide was the first phylloseptin discovered in the skin secretion of *P. vaillantii*, it was systematically named phylloseptin-PV1 (PPV1). The sequence has been deposited in the GenBank with the accession number: MT497983.

### Secondary Structure of Phylloseptin-PV1

**FIGURE 2:**
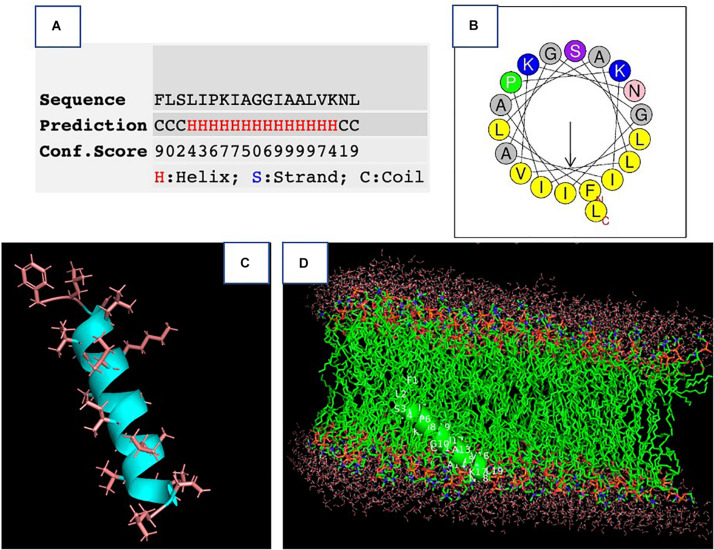
The prediction of the secondary structure of PPV1. **(A)** The possibility of a helical domain on PPV1 determined by the I-TASSER server. **(B)** The helical wheel projection of PPV1. **(C)** The predicted 3D conformation of PPV1 using the I-TASSER server. The side chains of amino acid residues are indicated. **(D)** The docking of PPV1 conformation from **(C)** with lipid bilayer using the Patchdock online server (Heller et al., 1993).

**FIGURE 3:**
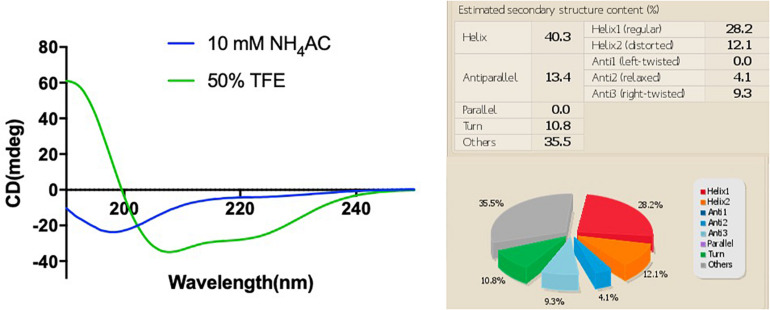
The CD spectra of 100 μM of PPV1 in both 10 mM NH_4_AC solution and in 50% TFE (2,2,2-trifluoroethanol)/10 mM NH_4_AC solution. The spectrum in the 50% TFE solution was analyzed by the BESTSEL online server.

The synthetic peptide was purified by RP-HPLC and its identity confirmed by MALDI-TOF mass spectrometry (Supplementary Figure 3). As online servers predicted, PPV1 was able to form an α-helical structure (Figure 2), which is consistent with the CD spectra using the membrane-mimicking 50% TFE solution (Figure 3). The calculated percentage of helical content based on the spectrum in 50% TFE solution is 40.3% However, it adopted a random coil structure in an aqueous environment. As the docking result showed, the hydrophobic N-terminal domain of PPV1 could bind with the fatty acid chain of phospholipid, while the C-terminal charged domain could bind with the negatively charged lipid heads. The physicochemical properties are showed in Supplementary Table 2.

### Antimicrobial and Antibiofilm Activities of PPV1

**FIGURE 4:**
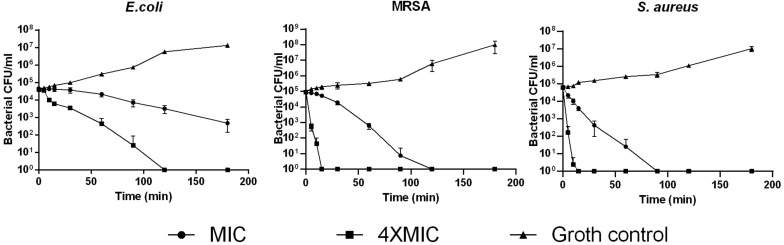
The time-killing curves of PPV1 against *E. coli*, *S. aureus*, and MRSA over 180 min, at the peptide concentration corresponding to 1 × MIC and 4 × MIC. The bacterial treated with broth only was used as growth control. The error bars represent the mean ± SD of nine replicates from three independent experiments.

Phylloseptin-PV1 showed broad-spectrum antimicrobial and antibiofilm activities against the tested microorganisms (Table 1). It showed more potent activity against Gram-positive bacteria and *C. albicans* than against Gram-negative bacteria. Particularly, PPV1 was not able to inhibit biofilm formation or eradicate established biofilms of *P. aeruginosa* and *K. pneumoniae.* Additionally, PPV1 demonstrated the antimicrobial activity against *S. aureus* strains isolated from the cystic fibrosis patients. As shown in Supplementary Table 3, the antimicrobial activity of PPV1 was not affected at high temperature. The time-killing curves demonstrated that PPV1 was capable of killing MRSA and *S. aureus* at corresponding MICs and fourfold MICs. While, it is only able to kill *E. coli* at fourfold MIC. The fourfold MICs of PPV1 can kill *S. aureus* and MRSA within 15 min, but it cost 90 and 120 min to kill them at onefold MICs, respectively. PPV1 killed *E. coli* more slowly than *S. aureus*, and it is not able to kill them at onefold MIC (Figure 4).

**TABLE 1:** The antimicrobial and antibiofilm activities of PPV1 against different microorganisms.

PPV1	MIC/μM	MBC/μM	MBIC/μM	MBEC/μM
*S. aureus* (NCTC 10788)	4	8	8	16
*S. aureus* (ATCC 6538)	2	4	4	8
*S. aureus* (B038 V1S1A)	8	16	32	64
*S. aureus* (B042 V2E1A)	8	16	16	64
MRSA (ATCC 12493)	4	8	8	16
*E. faecalis* (NCTC 12697)	32	32	64	256
*E. coli* (NCTC 10418)	32	64	32	64
*E. coli* (ATCC BAA-2340)	64	128	128	512
*E. coli* (ATCC CRM-8739)	128	256	256	>512
*P. aeruginosa* (ATCC 27853)	128	128	>512	>512
*P. aeruginosa* (ATCC CRM-9027)	256	>512	>512	>512
*P. aeruginosa* (B004 V2 S2 B)	>512	>512	>512	>512
*K. pneumoniae* (ATCC 43816)	64	128	>512	>512
*K. pneumoniae* (ATCC BAA-1705)	256	512	>512	>512
*K. pneumoniae* (ATCC BAA-2342)	128	256	>512	>512
*C. albicans* (NCYC 1467)	8	16	16	32

### Membrane Permeabilization by PPV1

**FIGURE 5:**
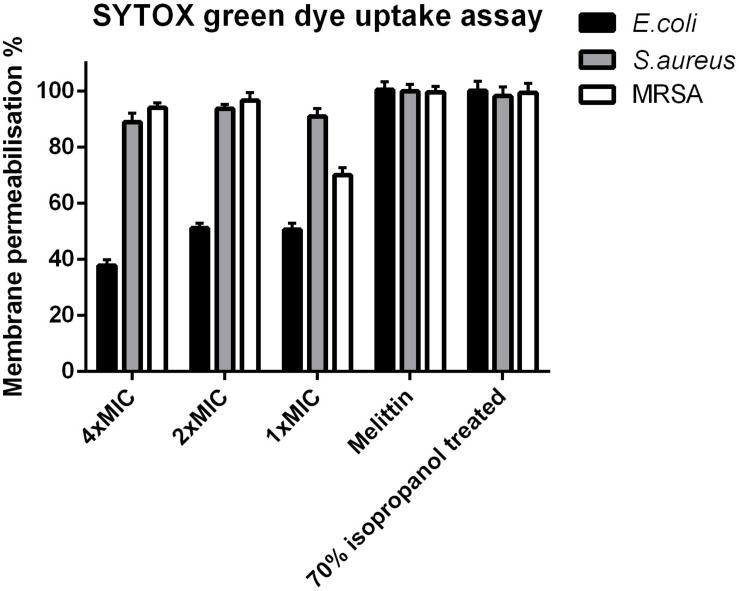
Cell membrane permeabilization effects of PPV1 on *E. coli*, *S. aureus*, and MRSA, detected by the uptake of SYTOX Green dye assay at peptide concentrations corresponding to 1 × MIC, 2 × MIC, and 4 × MIC. The bacteria without any treatment was employed as negative control. The 100% of membrane permeabilization effect was achieved by the bacterial cell that were treated by 70% isopropanol. A cytolytic peptide, Melittin (32 μM) was employed in this assay as well. The error bars represent the mean ± SD of nine replicates from three independent experiments.

Phylloseptin-PV1 permeabilized the cell membrane of *S. aureus* and MRSA as effectively as the known cytolytic peptide, melittin, but its potency was weaker than that of melittin against *E. coli* (Figure 5). PPV1 only permeabilized around 50% cell membrane on *E. coli* cells and this effect was slightly decreased at the high concentration.

### Cell Viability and Hemolysis Effects of PPV1

**FIGURE 6:**
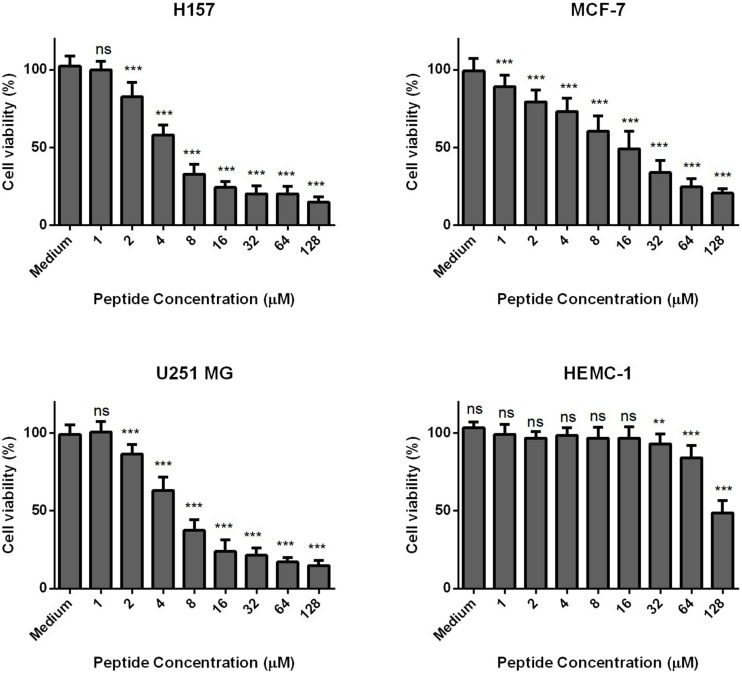
The antiproliferative effects of PPV 1 on the cell lines, MCF-7, H157, U-251 MG, and HMEC-1. The results are expressed as cell viability (%) at different peptide concentrations from 10^– 4^ M to 10^– 9^ M. Medium control represents the cells growing in the fresh medium without any treatment, which was employed as 100% cell viability. The data represents means ± SD of nine replicates from three independent experiments, and asterisks indicates *p*-values of each sample group compared with the medium group using one-way AONVA analysis: **p < 0.01; ***p < 0.001; ns, no significant difference.

**FIGURE 7:**
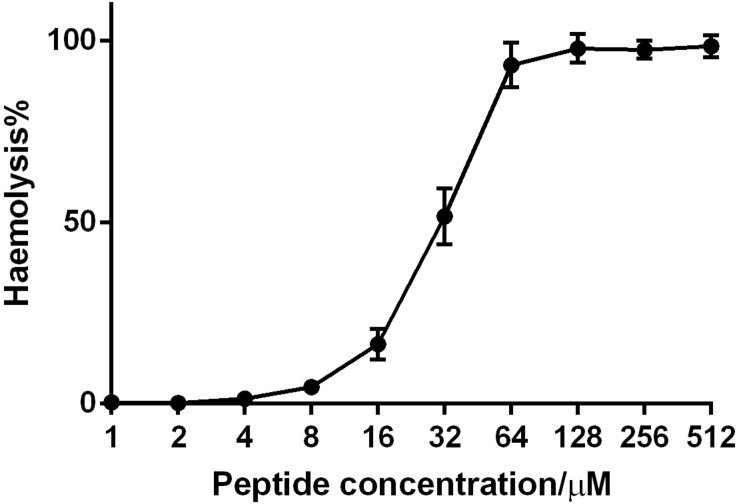
The hemolytic activity of PPV1 on horse erythrocytes. The 100% hemolysis was conducted using the treatment of the positive control, 1% TritonX-100. The erythrocytes that was resuspended in PBS was used as negative control. The error bars indicate the mean ± SD of 15 replicates from three independent experiments.

Phylloseptin-PV1 decreased the cell viability of selected cancer cell lines. It was more potent against MCF-7, H157, and U251MG (Figure 6), but less potent against HMEC-1. The IC_50_ values obtained against tested cell lines are shown in Supplementary Table 4. PPV1 induced the hemolysis of horse erythrocytes and caused less than 10% hemolysis at concentrations up to 8 μM (Figure 7) which was a higher concentration than observed MICs against *S. aureus* and MRSA.

### The Antimicrobial Activity of PPV1 on the Infected Mice

**FIGURE 8:**
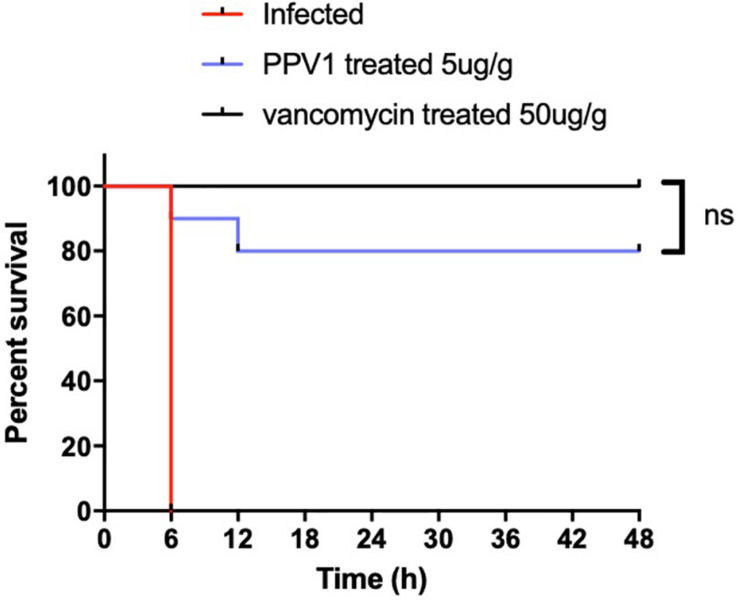
*In vivo* antimicrobial activity of PPV1 in the treatment of infected mice. Survival rate of female C57BL/6 J mice infected with *S. aureus* over 48 h following intraperitoneal injection of PPV1 at 5 μg/g every 12 h. PBS and vancomycin at 50 μg/g were employed as negative control and positive control, respectively. The significance of survival curves was analyzed by log-rank test in Prism software.

**FIGURE 9:**
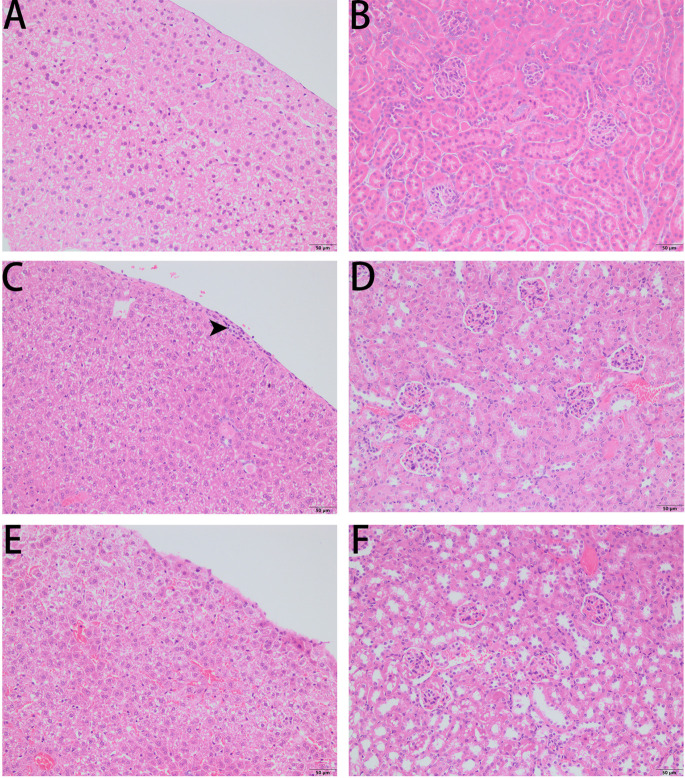
Liver histopathologic changes **(A,C,E)** and kidney histopathologic changes **(B,D,F)** of *S. aureus* infected C57BL/6 J mice following intraperitoneal injection of PPV1. **(A,B)** The control tissues were treated with PBS. **(C)** The liver from *S. aureus* infected mice revealed neutrophil cell infiltration in the liver capsular area (indicated by arrowhead). **(D)** While, there is no significant histopathologic changes to kidney from the infected mice. **(E)** With the treatment of PPV1 (5 μg/g), the liver capsule shows no inflammatory cell infiltration, and **(F)** kidney did not show any obvious changes. The magnification is ×200. The bar length represents 50 μm.

As PPV1 effectively inhibited the growth of *S. aureus in vitro* at the concentration without any hemolysis on horse blood cells. The *S. aureus* infected C57BL/6 J was employed as a model for investigation of the *in vivo* antimicrobial potency. As shown in Figure 8, PPV1 at 5 μg/g significantly ameliorated the survival rates of the mice with injection of *S. aureus* (ATCC 6538) and their survival rate was not significantly different to those that received vancomycin at 50 μg/g. According to the appearance of H&E-stained liver (Figure 9), PPV1 ameliorated neutrophil cell infiltration, caused by *S. aureus*. While, there is no obvious morphological changes among the kidney tissue. Additionally, PPV1 did not induce lysis of red blood cells or induce significant toxicity on liver and kidney as assessed by monitoring of functions (Table 2).

**TABLE 2:** Female C57BL/6 J mice infected with *S. aureus* were treated with PPV1 by intraperitoneal injection (right side) at a dose of 5 μg/g after infection.

	*S. aureus* + PPV1	*S. aureus*	PBS
RBC (×10^6^ cells/μL)	9.06 ± 0.92	8.96 ± 0.74	9.23 ± 0.23
WBC (×10^3^ cells/μL)	4.58 ± 1.35	4.68 ± 1.22	5.24 ± 0.87
HGB (g/DL)	13.4 ± 1.1	12.4 ± 1.07	13.66 ± 0.64
ALT (U/L)	23 ± 4	32 ± 3.8	28.75 ± 1.25
AST (U/L)	104 ± 20	158 ± 18.6	124.5 ± 19.5
ALP (U/L)	71.6 ± 9.4	79 ± 6.9	134.4 ± 10.5
CREAT (umol/L)	15.8 ± 1.8	12 ± 2.1	13.8 ± 1.8

*Data shown are the mean ± SD for testing performed on n = 10 mice. HGB, hemoglobin; ALT, alanine aminotransferase; AST, aspartate aminotransferase; ALKP, alkaline phosphatase; CREAT, creatinine.*

### *In vivo* Toxicity of PPV1

**FIGURE 10:**
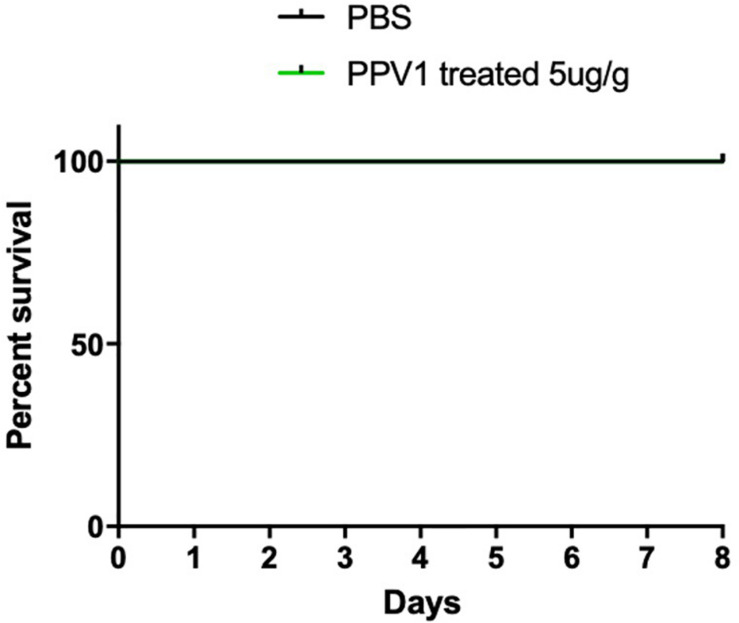
The mortality of CD-1 mice treated with PPV1. Survival rate of CD-1 mice up to 8 days following intraperitoneal injection with PPV1 at 5 μg/g twice daily, was recorded. The negative control group was treated with PBS alone. The significance of survival curves was analyzed by log-rank test in Prism software.

**FIGURE 11:**
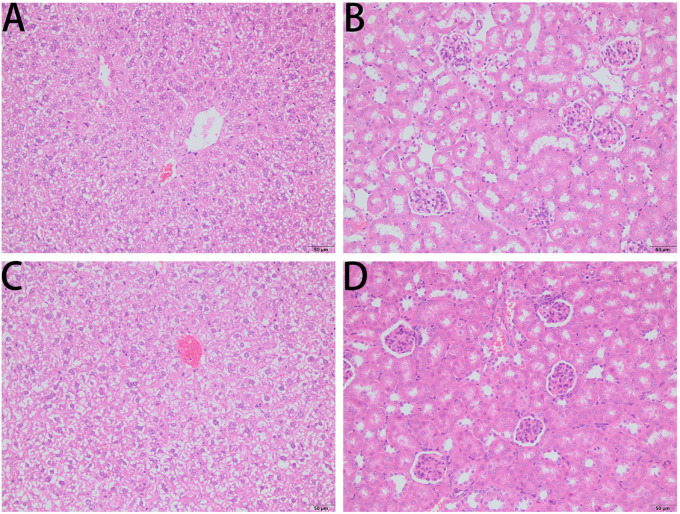
H&E-stained liver sections **(A,C)** and kidney sections **(B,D)** from a representative mouse from?10 treated with PBS **(A,B)** and PPV1 **(C,D)** at an intraperitoneal dose of 5 μg/g administered twice daily for 8 days, revealed normal tissue and cellular architecture. Both tissues did not demonstrate any histopathologic changes induced by the administration of PPV1 for 8 days. The magnification is ×200. The bar length represents 50 μm.

The survival rate of CD-1 mice up to 8-days with intraperitoneal injection twice daily with PPV1 was the same as that of the blank control (Figure 10). The H&E-stained liver and kidney sections and the blood tests performed, showed that PPV1 at 5 μg/g did not cause significant toxic effects in CD-1 mice (Figure 11 and Table 3).

**TABLE 3:** Male CD-1 mice were treated with PPV1 at 5 μg/g twice daily for 8 days by intraperitoneal injection.

	5 μg/g ip injection twice daily for 8 days	Blank	Normal range
RBC (× 10^6^ cells/μL)	8.69 ± 0.81	7.88 ± 0.62	7.0–10.1
WBC (×10^3^ cells/μL)	5.29 ± 0.64	4.86 ± 0.91	3.2–12.7
HGB (g/DL)	13.87 ± 0.63	13.14 ± 0.54	11.8–14.9
ALT (U/L)	24.8 ± 5.2	27.8 ± 0.8	28–64
AST (U/L)	83.4 ± 24.2	75 ± 19	47–120
ALP (U/L)	177 ± 26	184 ± 33	101–207
CREAT (umol/L)	14 ± 1	11.6 ± 2.6	0–30

*Data shown are the mean ± SD for testing performed on n = 10 mice. HGB, hemoglobin; ALT, alanine aminotransferase; AST, aspartate aminotransferase; ALKP, alkaline phosphatase; CREAT, creatinine.*

## Discussion

Bioactive peptides from the skin secretions of amphibians are supposed to be an important component of their immune systems. These peptides exhibit multifunctional activities, including the inhibition of Gram-negative and Gram-positive bacteria, fungi and some cancer cells (Wu et al., 2017). In addition, they also can be acquired readily in quantity by chemical synthesis, which is convenient for mass production and this may increase their potential to become new medicines against microbial infection and drug resistance (Huang et al., 2017).

Current research has shown that the skin secretions of Neotropical phyllomedusine frogs are one of the richest sources for discovery of novel antibacterial peptides (Amiche et al., 2008). In this study, a novel AMP, named PPV1, was discovered in the skin secretions of the leaf frog, *P. vaillantii*, and it showed different antimicrobial potencies against test microorganisms. As the results of MICs and MBCs shown, it exhibited potent bacteriostatic and bactericidal activities against Gram-positive bacteria and *C. albicans*, while, its overall antimicrobial activity against Gram-negative bacteria was weaker. This phenomenon can be often observed among the other members in phylloseptin family (Wu et al., 2017, 2019; Proaño-Bolaños et al., 2019). For instance, phylloseptin-L1 is able to inhibit the growth of *S. aureus* at the concentration of 8 μM, while it has no effect on *E. coli* (Conlon et al., 2007).

As it is widely recognized, the bacteria killing mechanisms of AMPs are associated with the membrane premeabilization effect that could be exerted through several vital characteristics, such as cationicity, hydrophobicity, amphipathicity, and secondary structures (Raheem and Straus, 2019). AMPs can attach on the surface of bacteria cells by electrostatic interaction and further pass over the cell wall to reach the target, cell plasma membrane. Accordingly, the capability of AMPs to traverse along the call wall could considerably affect their antimicrobial activity. With regards to Gram-negative bacteria, the outer membrane consists of a large proportion of lipopolysaccharides (LPS) which has an amphiphilic structure containing a lipid A, an O antigen, and a core oligosaccharide. It is proved that LPS in the outer membrane could inactivate some AMPs by inducing self-association or aggregations of peptides, as it is much more difficult for the oligomeric AMPs to translocate through the outer membrane (Mohanram and Bhattacharjya, 2014). Although there is no study to show the aggregation effect of phylloseptin peptides in LPS micelles directly, it could be deduced that phylloseptin may be inactivated by LPS the similar way as an AMP, temporin-TA (FLPLIGRVLSGIL-NH_2_) (Saravanan et al., 2013). The aromatic sidechain of its Phe residue at the N-terminus generates packing interaction with the hydrophobic sidechain at 4th and 5th positions, which could form an “anchor” domain. Subsequently, this domain of the helical temporin-TA could bind with the hydrophobic region at C-terminus (-GIL-) of another temporin-TA molecule to form the oligomer in the LPS micelles (Saravanan et al., 2013). As we can see from the sequence of PPV1, the FLSLIP- domain at N-terminus might also pack into the “anchor” conformation. Similarly, PPV1 might undergo the hydrophobic interaction between the “anchor” and the hydrophobic region, such as the -IAGGIAA- helical domain and the C-terminal Phe sidechain. On the other hand, bacteria possess different surface structure, such as pili, fimbriae, fibrillae, and capsule, which, to a certain extent, could also separate PPV1 from the cell membrane (Campos et al., 2004).

Generally, AMPs are considered to form transmembrane pores, including carpet, barrel-stave, and toroidal pore-type models when the peptides reach a concentration threshold on the cell membrane (Gaspar et al., 2013). As showed in Figure 2D, PPV1 could extensively interact with the acyl region of the phospholipids to disintegrate bacterial cell membrane (Raja et al., 2013). SYTOX green dye uptake assays also proved that the cell membranes were permeabilized by the treatment of PPV1. In fact, PPV1 showed a similar membrane permeabilization effect to melittin against Gram-positive bacteria, while it was less potent in disrupting the cell integrity of *E. coli*. The similar situation was observed in the time-killing assay, where PPV1 kills MRSA and *S. aureus* more rapidly and effectively than *E. coli*. It is consistent with the hypothesis that PPV1 might encounter difficulties in translocating or diffusing through the outer membranes of Gram-negative bacteria, which has also been reported for other AMPs (Hancock, 2001).

Biofilm is a special structure consisting of a multicellular community of microorganisms on surfaces, and this structure is believed to be responsible for more than 50% of all human infections (De la Fuente-Núñez et al., 2014), Problematically, it has already been proven to provide obvious resistance to various chemical, physical, and biological antimicrobial agents, and to cause many persistent infections (Guilhelmelli et al., 2013). PPV1 exhibited inhibitory activity against biofilm formation in *S. aureus* and MRSA at a relatively low MBIC, and it eradicated mature biofilm, which may imply a potential to become an effective agent for killing the sessile biofilms of *S. aureus* and MRSA on the surfaces of medical instruments (Ohara et al., 1998). Although, the mechanism of antibiofilm effect is not clear yet, the possible assumption could be deduced from their bacterial killing mechanisms that PPV1 might exert detergent-like effect to disperse the mature biofilm as well as killing the bacteria embedded in the biofilm (Dou et al., 2017).

Currently, cancer is one of the main causes of the loss of human life and known anti-cancer peptides usually share a similar α-helical or β-sheet structure with most AMPs (Hoskin and Ramamoorthy, 2008), which is why PPV1 was also screened for anti-cancer activity. The results showed that it did induce inhibition against the tested cancer cell lines. This selectivity may be due to the negative charge of cancer cell membranes, which differs from the membranes of normal mammalian cells (Schweizer, 2009). As a result, the positively charged peptides can bind to the cancer cell membranes, and undergo the same way as that they act on the bacteria cell membrane (Tornesello et al., 2020). In accordance with the cytotoxicity of PPV1 on HMEC-1 and erythrocytes, PPV1 inhibited around 50% cell growth at 100 μM and induced more than 50% hemolysis at 32 μM, which revealed the similar effect to the other phylloseptins (Conlon et al., 2007). According to Dathe et al. (1997), the cell membranes of erythrocytes are mainly composed of zwitterionic phosphatidylcholine and phosphatidylethanolamine, so the hemolytic activity may not closely relate to the net charge. However, hydrophobicity may be a significant factor, since the correlation between hydrophobicity and hemolytic activity has already been observed for some AMPs (Dathe et al., 1997). As a previous study has shown, the balance of hydrophobicity and net charge remarkably affected the selectivity and cytotoxicity of phylloseptins (Liu et al., 2019). Higher constitution of hydrophobic amino acids on the hydrophobic surface of amphipathic helical dipole could extensively enhance the hemolytic activity. Considering that PPV1 contains more than 50% hydrophobic amino acid residues, it may induce a potent hydrophobic interaction with zwitterionic cell membranes to induce hemolytic activity (Kondejewski et al., 1999).

Most members in the phylloseptin family exhibited considerable hemolytic activity (Raja et al., 2013; Liu et al., 2017; Wu et al., 2017), which is a factor that limited their potential for clinical applications. No or very low host cell cytotoxicity is considered as one of the most important criteria for utilization of AMPs. Herein, PPV1 induced mild hemolytic activity at the concentration against the growth of *S. aureus* (up to 16 μM), though it caused significant hemolytic activity. Therefore, we assumed that it might be able to treat with the infection by *S. aureus in vivo* without generate significant toxicity. Besides, there are no *in vivo* studies reported which have investigated the efficacy of phylloseptins so far. Therefore, considering the potent anti-staphylococcal effects of PPV1, an *in vivo* study using *S. aureus* infected C57BL/6 J mice, an ideal model for such studies, was performed to obtain reliable *in vivo* data for further evaluation of antimicrobial activity of PPV1 (Hohl, 2014). Data indicated that PPV1 exhibited significant potency against *S. aureus in vivo*, which almost approached the effect of vancomycin. Additionally, PPV1 could reduce neutrophil cell infiltration in the liver caused by *S. aureus*, which indicates that PPV1 may kill the bacteria in the peritoneal cavity directly so that the immune cells would not be recruited by the bacteria invaded into the liver capsule.

Interestingly, the concentration of PPV1 used in the *in vivo* model did not induce any toxicity toward liver and kidney after the 8-day intraperitoneal injection study and caused no apparent damage to red blood cells conflicting somewhat with data from our hemolysis assay. It is speculated that the red blood cells used in the hemolysis assay were derived from horse and stored for a period prior to the study, which could have made them more fragile. Another cause for this discrepancy could have been that the peptide did not directly contact the red blood cells following intraperitoneal injection, which the lethal effect could be four time higher by a non-systemical administration than the intravenous administration (Pini et al., 2007). Also the whole animal model provides a considerably more complex environment compared to that of a test tube with few components. Unlike to melittin, a cytolytic peptide from bee venom that produces significant hemolytic activity to red blood cells and toxicity *in vivo* (Ownby et al., 1997), PPV1 possesses relative weak amphipathicity that could prevent from the non-specific killing effect to the normal cells.

## Conclusion

In summary, a novel AMP, a member of the phylloseptin family, was found in the skin secretion of *P. vaillantii*, and this peptide demonstrated more effective antimicrobial activity against Gram-positive bacteria than Gram-negative bacteria. Especially, PPV1 exhibited more potent effect against *S. aureus*. The *in vivo* testing showed that PPV1 significantly reduced the mortality of *S. aureus* infected mice without severe toxicity. However, we admit that data for supporting such expectation is still preliminary, especially the considerable hemolytic activity of PPV1 could extensively compromise its potential clinical application. The further modification would be taken into consideration such as stapling the sequence to maintain the secondary structure to improve the cell selectivity (Mourtada et al., 2019), and encapsulation of AMPs within liposomes or nanoparticles (Ron-Doitch et al., 2016). Additionally, these data suggest that PPV1 exhibits an encouraging antimicrobial potential when applied by localized administration *in vivo*.

## Data Availability Statement

The datasets presented in this study can be found in online repositories. The names of the repository/repositories and accession number(s) can be found below: https://www.ncbi.nlm.nih.gov/genbank/, MT497983.

## Ethics Statement

The animal study was reviewed and approved by the United Kingdom Animal (Scientific Procedures) Act 1986, project license PPL 2694, issued by the Department of Health, Social Services and Public Safety, Northern Ireland, the IACUC of Queen’s University Belfast, and the Animal Care and Use Committee of Nanjing University of Chinese Medicine (ACU191002).

## Author Contributions

TC, MZ, and LW contributed conception and design of the study. YL, DS, and JW performed the peptide identification and molecular cloning. YL and CM synthesized the peptide molecules and wrote the first draft of the manuscript. YL and XC performed *in vitro* assays. XX contributed conception and design of the *in vivo* study. YL and JC performed the *in vivo* assays with mice. CS wrote sections of the manuscript. All authors contributed to manuscript revision, read and approved the submitted version.

## Conflict of Interest

The authors declare that the research was conducted in the absence of any commercial or financial relationships that could be construed as a potential conflict of interest.

## References

[B1] Amiche M. Ladram A. Nicolas P. (2008). A consistent nomenclature of antimicrobial peptides isolated from frogs of the subfamily *Phyllomedusinae*. *Peptides* 29 2074–2082. 10.1016/j.peptides.2008.06.017 18644413

[B2] Ansari S. Jha R. K. Mishra S. K. Tiwari B. R. Asaad A. M. (2019). Recent advances in *Staphylococcus aureus* infection: focus on vaccine development. *Infect. Drug Resist.* 12:1243. 10.2147/idr.s175014 31190912 PMC6526327

[B3] Campos M. A. Vargas M. A. Regueiro V. Llompart C. M. Albertí S. Bengoechea J. A. (2004). Capsule polysaccharide mediates bacterial resistance to antimicrobial peptides. *Infect. Immun.* 72 7107–7114. 10.1128/iai.72.12.7107-7114.2004 15557634 PMC529140

[B4] Chung P. Y. Khanum R. (2017). Antimicrobial peptides as potential anti-biofilm agents against multidrug-resistant bacteria. *J. Microbiol. Immunol. Infect.* 50 405–410. 10.1016/j.jmii.2016.12.005 28690026

[B5] Conlon J. M. Woodhams D. C. Raza H. Coquet L. Leprince J. Jouenne T. (2007). Peptides with differential cytolytic activity from skin secretions of the lemur leaf frog Hylomantis lemur (*Hylidae*: *Phyllomedusinae*). *Toxicon* 50 498–506. 10.1016/j.toxicon.2007.04.017 17561225

[B6] Dathe M. Wieprecht T. (1999). Structural features of helical antimicrobial peptides: their potential to modulate activity on model membranes and biological cells. *Biochim. Biophys. Acta Biomembr.* 1462 71–87. 10.1016/s0005-2736(99)00201-1 10590303

[B7] Dathe M. Wieprecht T. Nikolenko H. Handel L. Maloy W. L. MacDonald D. L. (1997). Hydrophobicity, hydrophobic moment and angle subtended by charged residues modulate antibacterial and haemolytic activity of amphipathic helical peptides. *FEBS Lett.* 403 208–212. 10.1016/s0014-5793(97)00055-0 9042968

[B8] De la Fuente-Núñez C. Reffuveille F. Haney E. F. Straus S. K. Hancock R. E. (2014). Broad-spectrum anti-biofilm peptide that targets a cellular stress response. *PLoS Pathog.* 10:e1004152. 10.1371/journal.ppat.1004152 24852171 PMC4031209

[B9] Dou X. Zhu X. Wang J. Dong N. Shan A. (2017). Novel design of heptad amphiphiles to enhance cell selectivity, salt resistance, antibiofilm properties and their membrane-disruptive mechanism. *J. Med. Chem.* 60 2257–2270. 10.1021/acs.jmedchem.6b01457 28230992

[B10] Dreyfus J. Yu H. Begier E. Gayle J. Olsen M. A. (2020). Incidence of *Staphylococcus aureus* infections after elective surgeries in US hospitals. *Clin. Infect. Dis.* 2020:ciaa913. 10.1093/cid/ciaa913 32634829

[B11] Gaiser R. A. Ayerra Mangado J. Mechkarska M. Kaman W. E. van Baarlen P. Conlon J. M. (2019). Selection of antimicrobial frog peptides and temporin-1 DR a analogs for treatment of bacterial infections based on their cytotoxicity and differential activity against pathogens. *Chem. Biol. Drug Design* 1–11. 10.1111/cbdd.13569 31102497 PMC7891380

[B12] Gao Y. Wu D. Wang L. Lin C. Ma C. Xi X. (2017). Targeted modification of a novel amphibian antimicrobial peptide from *Phyllomedusa tarsius* to enhance its activity against MRSA and microbial biofilm. *Front. Microbiol.* 8:628. 10.3389/fmicb.2017.00628 28469603 PMC5395648

[B13] Gao Y. Wu D. Xi X. Wu Y. Ma C. Zhou M. (2016). Identification and characterisation of the antimicrobial peptide, phylloseptin-PT, from the skin secretion of *Phyllomedusa tarsius*, and comparison of activity with designed, cationicity-enhanced analogues and diastereomers. *Molecules* 21:1667. 10.3390/molecules21121667 27918477 PMC6273899

[B14] Gaspar D. Veiga A. S. Castanho M. A. (2013). From antimicrobial to anticancer peptides. A review. *Front. Microbiol.* 4:294. 10.3389/fmicb.2017.00294 24101917 PMC3787199

[B15] Guilhelmelli F. Vilela N. Albuquerque P. Derengowski L. Silva-Pereira I. Kyaw C. (2013). Antibiotic development challenges: the various mechanisms of action of antimicrobial peptides and of bacterial resistance. *Front. Microbiol.* 4:353. 10.3389/fmicb.2017.00353 24367355 PMC3856679

[B16] Hancock R. E. (2001). Cationic peptides: effectors in innate immunity and novel antimicrobials. *Lancet Infect. Dis.* 1 156–164. 10.1016/s1473-3099(01)00092-5 11871492

[B17] Heller H. Schaefer M. Schulten K. (1993). Molecular dynamics simulation of a bilayer of 200 lipids in the gel and in the liquid crystal phase. *J. Phys. Chem.* 97 8343–8360. 10.1021/j100133a034

[B18] Hohl T. M. (2014). Overview of vertebrate animal models of fungal infection. *J. Immunol. Methods* 410 100–112. 10.1016/j.jim.2014.03.022 24709390 PMC4163114

[B19] Hoskin D. W. Ramamoorthy A. (2008). Studies on anticancer activities of antimicrobial peptides. *Biochim. Biophys. Acta Biomembr.* 1778 357–375. 10.1016/j.bbamem.2007.11.008 18078805 PMC2238813

[B20] Huang L. Chen D. Wang L. Lin C. Ma C. Xi X. (2017). Dermaseptin-ph: a novel peptide with antimicrobial and anticancer activities from the skin secretion of the south american orange-legged leaf frog, Pithecopus (*Phyllomedusa*) *Hypochondrialis*. *Molecules* 22:1805. 10.3390/molecules22101805 29064402 PMC6151546

[B21] Imperial I. C. Ibana J. A. (2016). Addressing the antibiotic resistance problem with probiotics: reducing the risk of its double-edged sword effect. *Front. Microbiol.* 7:1983. 10.3389/fmicb.2017.01983 28018315 PMC5156686

[B22] Kondejewski L. H. Jelokhani-Niaraki M. Farmer S. W. Lix B. Kay C. M. Sykes B. D. (1999). Dissociation of antimicrobial and hemolytic activities in cyclic peptide diastereomers by systematic alterations in amphipathicity. *J. Biol. Chem.* 274 13181–13192. 10.1074/jbc.274.19.13181 10224074

[B23] Kwiecinski J. M. Horswill A. R. (2020). *Staphylococcus aureus* bloodstream infections: pathogenesis and regulatory mechanisms. *Curr. Opin. Microbiol.* 53 51–60. 10.1016/j.mib.2020.02.005 32172183 PMC7244392

[B24] Leite J. R. S. Silva L. P. Rodrigues M. I. S. Prates M. V. Brand G. D. Lacava B. M. (2005). *Phylloseptins*: a novel class of anti-bacterial and anti-protozoan peptides from the *Phyllomedusa genus*. *Peptides* 26 565–573. 10.1016/j.peptides.2004.11.002 15752569

[B25] Liu J. Wu Q. Li L. Xi X. Wu D. Zhou M. (2017). Discovery of phylloseptins that defense against gram-positive bacteria and inhibit the proliferation of the non-small cell lung cancer cell line, from the skin secretions of *Phyllomedusa* frogs. *Molecules* 22:1428. 10.3390/molecules22091428 28850103 PMC6151776

[B26] Liu Y. Du Q. Ma C. Xi X. Wang L. Zhou M. (2019). structure-activity relationship of an antimicrobial peptide, *Phylloseptin*-Pha: balance of hydrophobicity and charge determines the selectivity of bioactivities. *Drug Design Dev. Ther.* 13:447. 10.2147/dddt.s191072 30774309 PMC6350648

[B27] Louis-Jeune C. Andrade-Navarro M. A. Perez-Iratxeta C. (2012). Prediction of protein secondary structure from circular dichroism using theoretically derived spectra. *Proteins Struct. Funct. Bioinform.* 80 374–381. 10.1002/prot.23188 22095872

[B28] McGuinness W. A. Malachowa N. DeLeo F. R. (2017). Focus: infectious diseases: vancomycin resistance in *Staphylococcus aureus*. *Yale J. Biol. Med.* 90:269. PMC5482303 28656013

[B29] Mohanram H. Bhattacharjya S. (2014). Resurrecting inactive antimicrobial peptides from the lipopolysaccharide trap. *Antimicrob. Agents Chemother.* 58 1987–1996. 10.1128/aac.02321-13 24419338 PMC4023739

[B30] Molchanova N. Hansen P. R. Franzyk H. (2017). Advances in development of antimicrobial peptidomimetics as potential drugs. *Molecules* 22:1430. 10.3390/molecules22091430 28850098 PMC6151827

[B31] Mourtada R. Herce H. D. Yin D. J. Moroco J. A. Wales T. E. Engen J. R. (2019). Design of stapled antimicrobial peptides that are stable, nontoxic and kill antibiotic-resistant bacteria in mice. *Nat. Biotechnol.* 37 1186–1197. 10.1038/s41587-019-0222-z 31427820 PMC7437984

[B32] Ohara T. Itoh Y. Itoh K. (1998). Ultrasound instruments as possible vectors of *Staphylococcal* infection. *J. Hosp. Infect.* 40 73–77. 10.1016/s0195-6701(98)90028-7 9777525

[B33] Ownby C. L. Powell J. R. Jiang M. S. Fletcher J. E. (1997). Melittin and phospholipase A2 from bee (*Apis mellifera*) venom cause necrosis of murine skeletal muscle in vivo. *Toxicon* 35 67–80. 10.1016/s0041-0101(96)00078-5 9028010

[B34] Pini A. Giuliani A. Falciani C. Fabbrini M. Pileri S. Lelli B. (2007). Characterization of the branched antimicrobial peptide M6 by analyzing its mechanism of action and in vivo toxicity. *J. Pept. Sci.* 13 393–399. 10.1002/psc.858 17486663

[B35] Proaño-Bolaños C. Blasco-Zúñiga A. Almeida J. R. Wang L. Llumiquinga M. A. Rivera M. (2019). Unravelling the skin secretion peptides of the gliding leaf frog, *Agalychnis spurrelli* (Hylidae). *Biomolecules* 9:667. 10.3390/biom9110667 31671555 PMC6920962

[B36] Radek K. Gallo R. (2007). Antimicrobial peptides: natural effectors of the innate immune system. *Semin. Immunopathol.* 29 27–43. 10.1007/s00281-007-0064-5 17621952

[B37] Raheem N. Straus S. K. (2019). Mechanisms of action for antimicrobial peptides with multiple biological functions. *Front. Microbiol.* 10:2866 10.3389/fmicb.2017.02866 PMC6927293 31921046

[B38] Raja Z. Andre S. Piesse C. Sereno D. Nicolas P. Foulon T. (2013). Structure, antimicrobial activities and mode of interaction with membranes of bovel phylloseptins from the painted-belly leaf frog, *Phyllomedusa sauvagii*. *PLoS One* 8:e70782 10.1371/journal.ppat.070782 PMC3742671 23967105

[B39] Resende J. M. Moraes C. M. Prates M. V. Cesar A. Almeida F. C. Mundim N. C. (2008). Solution NMR structures of the antimicrobial peptides phylloseptin-1,-2, and-3 and biological activity: the role of charges and hydrogen bonding interactions in stabilizing helix conformations. *Peptides* 29 1633–1644. 10.1016/j.peptides.2008.06.022 18656510

[B40] Ron-Doitch S. Sawodny B. Kühbacher A. David M. M. N. Samanta A. Phopase J. (2016). Reduced cytotoxicity and enhanced bioactivity of cationic antimicrobial peptides liposomes in cell cultures and 3D epidermis model against HSV. *J. Control. Rel.* 229 163–171. 10.1016/j.jconrel.2016.03.025 27012977

[B41] Saravanan R. Joshi M. Mohanram H. Bhunia A. Mangoni M. L. Bhattacharjya S. (2013). NMR structure of temporin-1 ta in lipopolysaccharide micelles: mechanistic insight into inactivation by outer membrane. *PLoS One* 8:e72718 10.1371/journal.ppat.072718 PMC3767682 24039798

[B42] Schweizer F. (2009). Cationic amphiphilic peptides with cancer-selective toxicity. *Eur. J. Pharmacol.* 625 190–194. 10.1016/j.ejphar.2009.08.043 19835863

[B43] Stiefel P. Rosenberg U. Schneider J. Mauerhofer S. Maniura-Weber K. Ren Q. (2016). Is biofilm removal properly assessed? Comparison of different quantification methods in a 96-well plate system. *Appl. Microbiol. Biotechnol.* 100 4135–4145. 10.1007/s00253-016-7396-9 26923144 PMC4824840

[B44] Timmons P. B. O’Flynn D. Conlon J. M. Hewage C. M. (2019). Structural and positional studies of the antimicrobial peptide brevinin-1BYa in membrane-mimetic environments. *J. Pept. Sc.* 25:e3208. 10.1002/psc.3208 31721374

[B45] Tornesello A. L. Borrelli A. Buonaguro L. Buonaguro F. M. Tornesello M. L. (2020). Antimicrobial peptides as anticancer agents: functional properties and biological activities. *Molecules* 25:2850. 10.3390/molecules25122850 32575664 PMC7356147

[B46] Tyler M. J. Stone D. J. Bowie J. H. (1992). A novel method for the release and collection of dermal, glandular secretions from the skin of frogs. *J. Pharmacol. Toxicol. Methods* 28 199–200. 10.1016/1056-8719(92)90004-k 1296824

[B47] Wan Y. Ma C. Zhou M. Xi X. Li L. Wu D. (2015). Phylloseptin-PBa—A novel broad-spectrum antimicrobial peptide from the skin secretion of the peruvian purple-sided leaf frog (*Phyllomedusa baltea*) which exhibits cancer cell cytotoxicity. *Toxins* 7 5182–5193. 10.3390/toxins7124878 26633506 PMC4690128

[B48] Wu D. Gao Y. Wang L. Xi X. Wu Y. Zhou M. (2016). A combined molecular cloning and mass spectrometric method to identify, characterize, and design frenatin peptides from the skin secretion of litoria infrafrenata. *Molecules* 21:1429. 10.3390/molecules21111429 27792198 PMC6273206

[B49] Wu X. Pan J. Wu Y. Xi X. Ma C. Wang L. (2017). PSN-PC: a novel antimicrobial and anti-biofilm peptide from the skin secretion of *Phyllomedusa-camba* with cytotoxicity on human lung cancer cell. *Molecules* 22:1896. 10.3390/molecules22111896 29112170 PMC6150266

[B50] Wu Y. Wang L. Zhou M. Chen T. Shaw C. (2019). Phylloseptin-PBa1, -PBa2, -PBa3: three novel antimicrobial peptides from the skin secretion of Burmeister’s leaf frog (*Phyllomedusa burmeisteri*). *Biochem. Biophys. Res. Commun.* 509, 664–673. 10.1016/j.bbrc.2018.12.156 30612735

[B51] Yang N. Li L. Wu D. Gao Y. Xi X. Zhou M. (2016). Discovery of novel bacterial cell-penetrating *Phylloseptins* in defensive skin secretions of the South American hylid frogs, *Phyllomedusa duellmani* and *Phyllomedusa coelestis*. *Toxins* 8:255. 10.3390/toxins8090255 27589802 PMC5037481

[B52] Ying Y. Wang H. Xi X. Ma C. Liu Y. Zhou M. (2019). Design of N-terminal derivatives from a novel dermaseptin exhibiting broad-spectrum antimicrobial activity against isolates from cystic fibrosis patients. *Biomolecules* 9:646. 10.3390/biom9110646 31653005 PMC6920804

[B53] Zasloff M. (2019). Antimicrobial peptides of multicellular organisms: my perspective. *Antimicrob. Pept.* 1117 3–6. 10.1007/978-981-13-3588-4_1 30980349

[B54] Zhang R. Zhou M. Wang L. McGrath S. Chen T. Chen X. (2010). Phylloseptin-1 (PSN-1) from *Phyllomedusa sauvagei* skin secretion: a novel broad-spectrum antimicrobial peptide with antibiofilm activity. *Mol. Immunol.* 47 2030–2037. 10.1016/j.molimm.2010.04.010 20451254

[B55] Zhou X. Shi D. Zhong R. Ye Z. Ma C. Zhou M. (2019). Bioevaluation of ranatuerin-2Pb from the frog skin secretion of *Rana pipiens* and its truncated analogues. *Biomolecules* 9:249. 10.3390/biom9060249 31242693 PMC6627226

