# Supplementary material for: A Novel Amphibian Antimicrobial Peptide, Phylloseptin-PV1, Exhibits Effective Anti-staphylococcal Activity Without Inducing Either Hepatic or Renal Toxicity in Mice
Source: Front Microbiol. 2020 Oct 26;11:565158. doi: 10.3389/fmicb.2020.565158 (PMC7649123; doi:10.3389/fmicb.2020.565158)
Supplement: Supplementary file 1 [file Data_Sheet_1.PDF]

Supplementary Material

1.1 Supplementary Figures

```
      M  A  F  L   K  K  S   L  F  L   V  L  F  L   G  L  V
  1  ATGGCTTTCT TGAAGAAATC TCTTTTCCTT GTACTATTCT TTGGATTGGT
     TACCGAAAGA ACTTCTTTAG AGAAAAGGAA CATGATAAGA AACCTAACCA
       S  L  S   I  C  E  E   E  K  R   E  T  E   E  K  E
 51  TTCCCTTTCC ATCTGTGAAG AAGAGAAAAG AGAGACTGAA GAGAAAGAAA
     AAGGGAAAGG TAGACACTTC TTCTCTTTTC TCTCTGACTT CTCTTTCTTT
     N  D  Q  E   E  D  D   K  S  E  E   K  R  F   L  S  L
101  ATGATCAAGA GGAAGATGAT AAAAGTGAAG AGAAGAGATT CCTCAGCCTA
     TACTAGTTCT CCTTCTACTA TTTTCACTTC TCTTCTCTAA GGAGTCGGAT
       I  P  K  I   A  G  G   I  A  A   L  V  K  N   F  G  *
151  ATACCAAAGA TAGCAGGTGG AATAGCTGCT CTTGTTAAAA ACTTAGGTTA
     TATGGTTTCT ATCGTCCACC TTATCGACGA GAACAATTTT TGAATCCAAT

201  ATACAATGTA ACATTTCATA ACTCTAAGGA GCACAATTAT CAATAATTGT
     TATGTTACAT TGTAAAGTAT TGAGATTCCT CGTGTTAATA GTTATTAACA
251  TCTCAAAATA CATTAAAGCA TATTTAACCA ACAAAAAAAA AAAAAAAAAA
     AGAGTTTTAT GTAATTTCGT ATAAATTGGT TGTTTTTTTT TTTTTTTTTT
301  AAAAAAAAAA
     TTTTTTTTTT
```

Supplementary Figure 1. Nucleotide sequence and corresponding translated open reading frame amino acid sequence of the biosynthetic peptide precursor of phylloseptin-PV1 encoded by cDNA cloned from *Phyllomedusa vaillantii* skin secretion. The putative signal peptide is doubled-underlined, the mature peptide is single-underlined, and the stop codon is indicated by an asterisk.

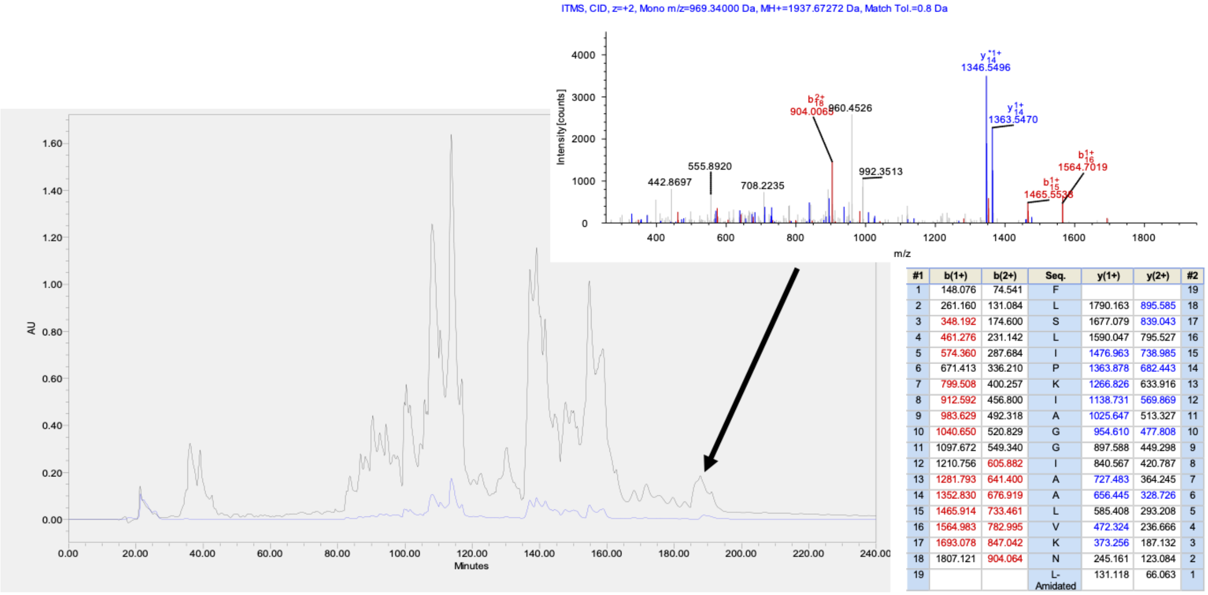

#1	b(1+)	b(2+)	Seq.	y(1+)	y(2+)	#2
1	148.076	74.541	F			19
2	261.160	131.084	L	1790.163	895.585	18
3	348.192	174.600	S	1677.079	839.043	17
4	461.276	231.142	L	1590.047	795.527	16
5	574.360	287.684	I	1476.963	738.985	15
6	671.413	336.210	P	1363.878	682.443	14
7	799.508	400.257	K	1266.826	633.916	13
8	912.592	456.800	I	1138.731	569.869	12
9	983.629	492.318	A	1025.647	513.327	11
10	1040.650	520.829	G	954.610	477.808	10
11	1097.672	549.340	G	897.588	449.298	9
12	1210.756	605.882	I	840.567	420.787	8
13	1281.793	641.400	A	727.483	364.245	7
14	1352.830	676.919	A	656.445	328.726	6
15	1465.914	733.461	L	585.408	293.208	5
16	1564.983	782.995	V	472.324	236.666	4
17	1693.078	847.042	K	373.256	187.132	3
18	1807.121	904.064	N	245.161	123.084	2
19			L-Amidated	131.118	66.063	1

Supplementary Figure 2. The RP-HPLC chromatogram of the skin secretion of *Phyllomedusa vaillantii* with dual wavelength detection (grey: 214 nm; blue: 280 nm), and the MS/MS fragmentation analysis of phylloseptin-PV1. MS/MS data was mapped against the cDNA encoded biosynthetic precursors using Sequest algorithm. The MS/MS spectrum showed in the upper right hand corner demonstrated the presence of phylloseptin-PV1 as well as the C-terminal amide. The retention time of phylloseptin-PV1 is indicated by the arrow. The observed b and y fragment ions from phylloseptin-PV1 are represented in the table.

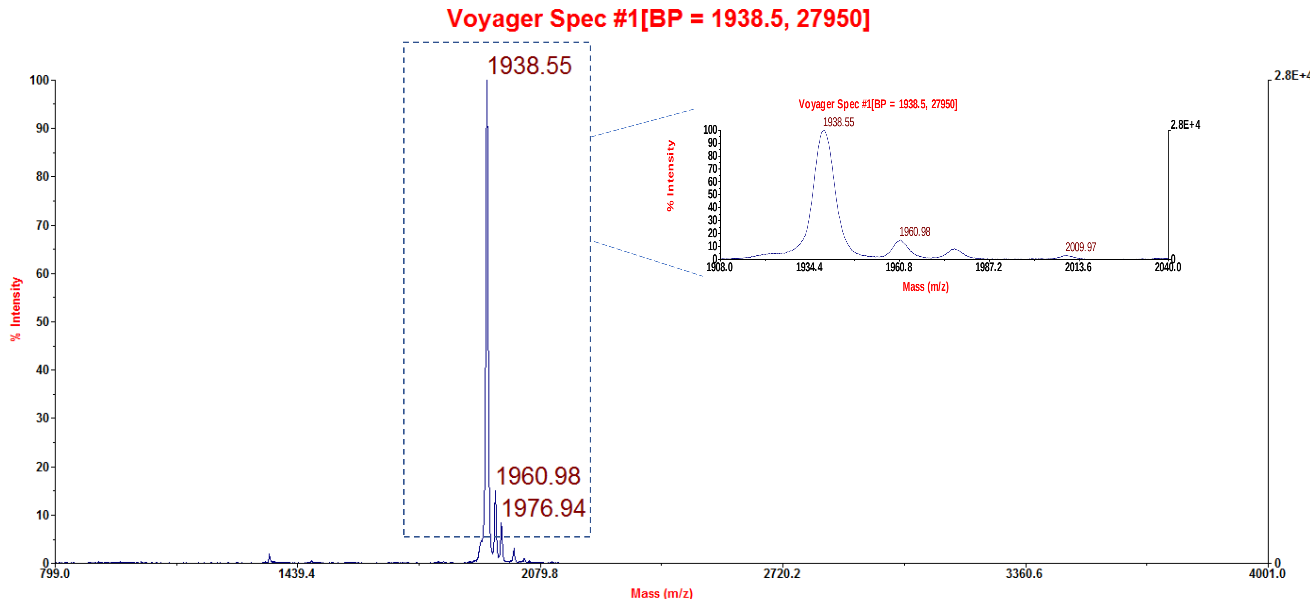

Supplementary Figure 3. The MALDI-TOF mass spectrum of the RP-HPLC purified phylloseptin-PV1 from the synthetic peptide. The observed average atomic mass of single-charged peptide ion $[M+H]^+$ is 1938.55 Da. Alpha-cyano-4-hydroxycinnarmic acid (CHCA) was employed as the matrix.

1.2 Supplementary Tables

Supplementary Table 1. The antibiotic resistance profiles of the clinic isolates from the cystic fibrosis patients. R represents that the strain is resistant to the tested antibiotics.

	**Amoxicllin**	**Azithromycin**	**Ceftazidime**	**Chloramphenicol**	**Clindamycin**	**Co-amoxiclav**	**Doxycycline**	**Meropenem**	**Metronidazole**	**Tobramycin**	**Colistin**	**Ciprofloxacin**
P. aeruginosa **B004 V2 S2 B**	R	R	R		R	R	R		R			R
S. aureus **BO38 V1S1A**	R	R	R				R		R	R	R	
S. aureus **BO42 V2E1 A**	R	R	R		R	R		R	R		R	R

Supplementary Table 2. Physicochemical properties of PPV1

Peptide	Secondary Structure	Hydrophobicity <H>	Hydrophobic moment <μH>	Charge	Helix %
**PPV1**	α-helix	0.697	0.569	2	40.3

Supplementary Table 3. MICs of PPV1 against *S.aureus* and MRSA at different temperatures.

Temperature	20℃	40℃	60℃	80℃	100℃
**MIC of *S.aureus*/μM**	4	4	4	4	4
**MIC of MRSA/μM**	4	4	4	4	4

Supplementary Table 4. IC_{50} values of PPV1 against tested cell lines.

IC_{50} (μM)	MCF-7	H157	U-251 MG	HMEC-1
**PPV1**	14.4	6.41	7.22	230.3
